# A novel locus for X-linked congenital cataract on Xq24

**Published:** 2008-04-18

**Authors:** Jamie E. Craig, Kathryn L. Friend, Jozef Gecz, Kate M Rattray, Mark Troski, David A. Mackey, Kathryn P. Burdon

**Affiliations:** 1 Department of Ophthalmology, Flinders University, Adelaide, SA, Australia; 2 Department of Genetic Medicine, Women’s and Children’s Hospital, Adelaide, SA, Australia; 3 Departments of Paediatrics and Molecular Biosciences, University of Adelaide, Adelaide, SA, Australia; 4 Centre for Eye Research Australia, University of Melbourne, Royal Victorian Eye and Ear Hospital, Melbourne, Australia

## Abstract

**Purpose:**

This study aimed to map the genetic locus responsible for a novel X-linked congenital cataract phenotype.

**Methods:**

A large three-generation family with lamellar and nuclear cataract in five affected males was identified. Linkage analysis was conducted by genotyping X-chromosome specific microsatellite markers at an average spacing of 5 cM. Analysis was conducted using the LINKAGE package under an X-linked recessive model.

**Results:**

A linkage was detected on Xq24 with the maximum LOD score of 2.53 at θ=0 for DXS1001. The minimal region was defined as 11.5 Mb between markers DXS8055 and DXS8009 through critical recombination events in multiple individuals.

**Conclusions:**

A gene causing this novel congenital cataract phenotype is located on the long arm of the X chromosome.

## Introduction

Congenital cataract is a heterogeneous group of disorders consisting of the presence of unilateral or bilateral cataract at or soon after birth. The term also often encompasses familial forms of juvenile cataract where the opacity is not detectable at birth but develops during childhood and is often progressive. The most common mode of inheritance for congenital and juvenile cataract is autosomal dominant. However, several instances of X-linked inheritance have been described [1-3].

Early literature regarding non-syndromic congenital cataract linked to the X chromosome was not convincing. However, several reports now indicate that X-linked cataract is a real phenomenon. Fraccaro et al. [1] described a five-generation pedigree in which the males had bilateral total nuclear cataract. No male to male transmission was observed, although only one affected male had offspring. Females also displayed a detectable phenotype of opacity of the posterior Y sutures while maintaining normal vision until their forties. This family demonstrated linkage to the Xg blood group locus on chromosome Xp22.33. In 1969, Krill et al. [2] described a three-generation pedigree also with total cataract in males and Y sutural opacity in females. No genetic study of this family was conducted. However, the absence of male to male transmission and the milder phenotype in females suggested X-linked inheritance. This disorder is now referred to in the Online Mendelian Inheritance in Man database as “Cataract, total congenital with posterior sutural opacities in heterozygotes” (OMIM 302200).

More recently, Francis et al. [3] described a five-generation pedigree with X-linked congenital cataract, again consisting of a total opacity in the males with all affected males requiring surgery in the first few weeks of life. In females, the phenotype was described as fan-shaped, nuclear, and slowly progressive. This somewhat different phenotype in heterozygotes compared with other reports suggests genetic heterogeneity among X-linked cataract pedigrees. The disease gene in this family was also mapped to Xp22 and has been refined to a 3.2 Mb region [4]. Interestingly, several of the males in this family also exhibited cardiac anomalies, suggesting the involvement of a gene regulating development of multiple tissues [3].

**Figure 1:**
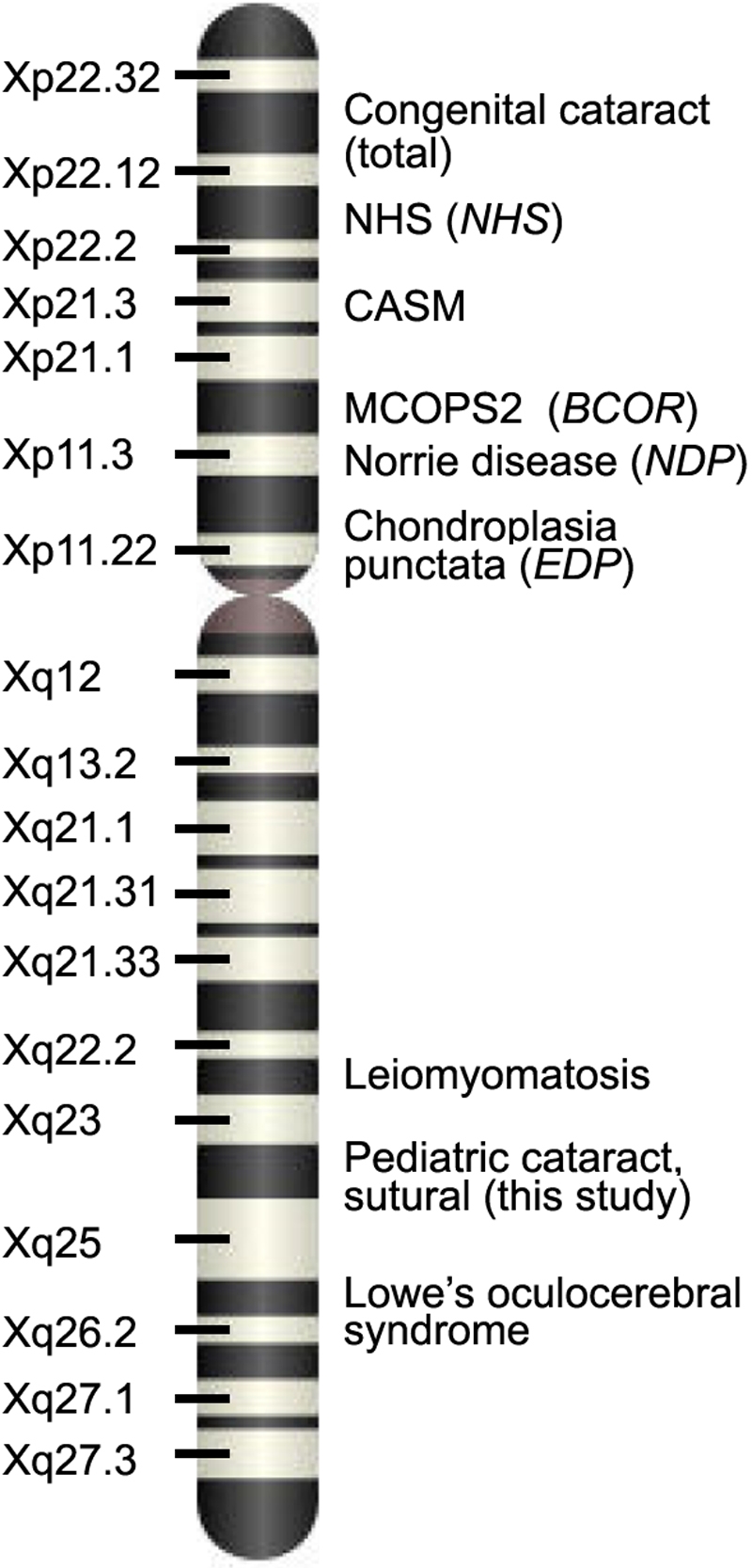
Ideogram of the X chromosome. Approximate location of mapped X-linked syndromes involving cataract are indicated. The locus mapped in this study does not overlap with previously described X-linked cataract loci.

Many syndromes, of which several are X-linked, have been described where cataract is a prominent feature (Figure 1). Several reports have been made of families with hereditary X-linked microphthalmia or microcornea and cataract (OMIM 302300), although no detailed mapping of this syndrome has been reported [5,6]. Gorlin et al. [7] present a review describing a syndrome consisting of ocular defects including microphthalmia and cataract as well as facial, cardiac, and dental abnormalities. Now named microphthalmia, syndromic 2 (MCOPS2, OMIM 300166), this syndrome is caused by mutations to the *BCOR* gene on Xp11.4 [8]. Other syndromic cataracts mapping to Xp include chondrodysplasia punctata 2 (Xp11.23-p11.22; OMIM 302960), which is caused by *EBP* gene mutations [9]; cataracts, ataxia, short stature, and mental retardation (CASM; Xpter-q13.1; OMIM 300619) [10]; Norrie Disease (Xp11.3, OMIM 310600), which is caused by mutations in the *NDP* gene [11]; and Nance-Horan syndrome (Xp22.13, OMIM 302350), which is caused by mutations in the *NHS* gene [12]. On the long arm of the X chromosome, syndromes involving cataract include Lowe Oculocerebral syndrome (Xq26.1, OMIM 309000) and Leiomyomatosis, esophageal, and vulval with nephropathy (Xq22.3, OMIM 308940), caused by a contiguous gene deletion involving two collagen genes [13]. There are several additional features accompanying cataract in all these syndromes including mental retardation, microphthalmia, dental abnormalities, dysmorphism, short stature, and esophageal tumors.

In this report, we describe a three-generation pedigree with X-linked congenital cataract in which the females do not exhibit any form of lens opacity. Affected males also display a range of other subtle dysmorphic features co-segregating with the cataract. We have demonstrated linkage of this disorder to Xq24, which is a novel locus for X-linked cataract, highlighting the genetic heterogeneity of all forms of congenital cataract including the X-linked forms.

## Methods

Male first cousins were referred to the same ophthalmologist for cataract surgery as teenagers and were noted to have similar unusual lens morphology and subtle dysmorphic facies. The pedigree was further investigated with clinical examinations of 22 individuals in three generations revealing a total of five affected males and a segregation pattern consistent with X-linked recessive inheritance. All participants gave written informed consent, and all protocols were approved by the Human Research Ethics Committees of Flinders University, Adelaide, Australia and the Royal Victorian Eye and Ear Hospital, Melbourne, Australia and adhered to the tenets of the Declaration of Helsinki.

**Figure 2:**
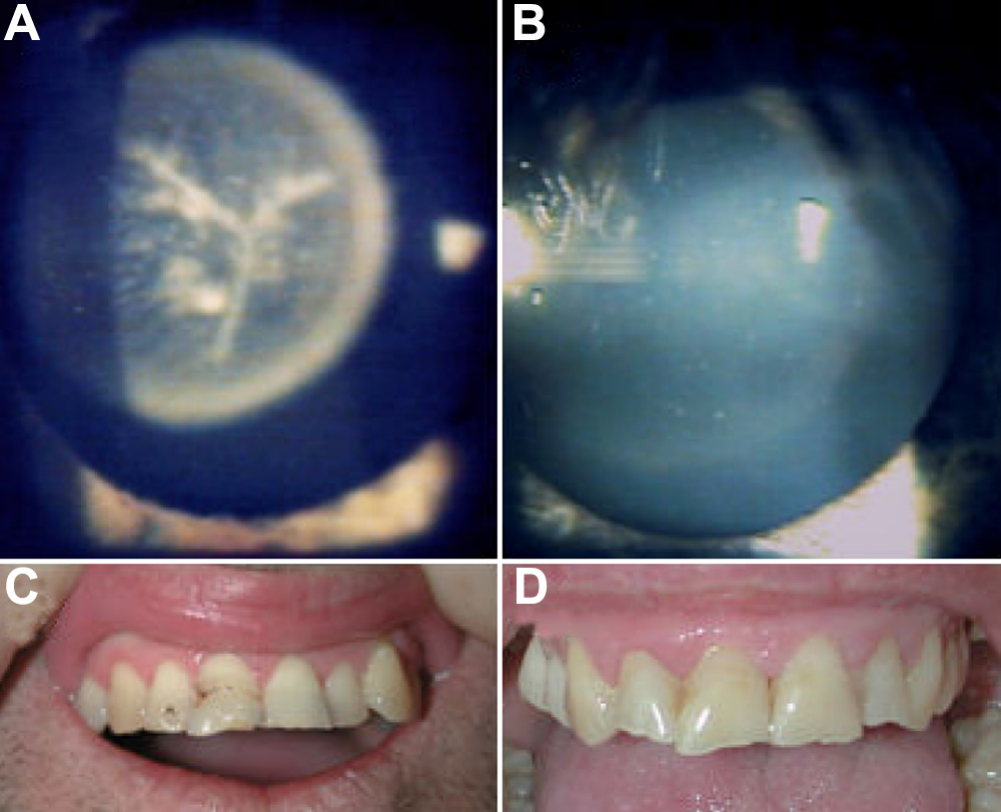
Cataract and dental phenotypes in affected males. **A** and **B:** Cataract in patients IV:3 and IV:15 shows the sutural cataract with nuclear lamellar involvement, which is present in all five affected males. **C** and **D**: The dentition in patients III:8 and III:12 shows the pyramidal incisors present in two patients.

DNA was extracted from whole blood using the QIAamp DNA blood maxi kit (Qiagen Pty Ltd, Doncaster, VIC, Australia) from all 22 available family members. A 5 cM linkage scan of the X chromosome was conducted with genotyping of microsatellite markers at the Australian Genome Research Facility (Melbourne, VIC, Australia) using Linkage Mapping set v2.5 (Applied Biosystems, Foster City, CA). Additional fine mapping markers, DXS8098 and DXS8057, were identified from publicly available databases (UCSC genome browser). A novel marker, CA-AC004000, was obtained by searching the reference sequence of the clone, AC004000, for CA repeats. Primers to amplify the region were designed in Primer3, forward: 5′- CTC TTT TGG TGT TAG GCC AGA-3′ and reverse: 5′- TCT TGG GGA AAG TGA TCC TG-3′. The forward primer was fluorescently labeled, and the allele size was detected on an ABI PRISM 3100 Genetic Analyzer (Applied Biosystems). The allele sizes were observed to be between 215 and 226 base pairs. Two-point linkage analysis was conducted using the MLINK component of the LINKAGE package [14]. The trait was modeled as an X-linked recessive trait with complete penetrance in homozygotes and hemizygous males and with a disease gene frequency of 0.0001. All females were set as unaffected regardless of known carrier status for the recessive model. Allele frequencies were set as equal for all markers. Haplotypes were manually reconstructed. Multipoint analysis was conducted in the X-linked version of the MERLIN package (MINX) [15]. The model used was identical to the two-point analysis. Map distances were taken from the Genethon map.

## Results

### Clinical description

The index case (IV:3) had bilateral cataracts diagnosed at the age of four years with slow progression requiring surgery at the age of 16 years. The lens opacity consisted of diffuse lamellar and nuclear opacification with prominent sutural involvement and white dots (Figure 2A). A male cousin (IV:15) had similar cataracts also requiring surgery at age 17 (Figure 2B). Three maternal uncles were also found to be affected (III:4, III:8, and III:12). One remains unoperated with milder lamellar cataracts (no sutural involvement). Obligate female carriers had no clinically significant lens opacity, although on close examination some small “salt grains” were present in III:2. Two affected males (III:8 and III:12) had pyramidal incisors of unusual appearance. (Figure 2C,D). Individual III:4 was edentulous with all teeth removed by age 25. All other affected males had dental crowding and jaw misalignment requiring extensive orthodontic work. However, due to the common occurrence of dental crowding in the general population, the importance of this feature is uncertain. Subtle dysmorphic features including high arched palate, long narrow face, and prominent ears were also observed in some of the males (Table 1).

**Table 1:** Clinical features of the five affected males.

**Clinical features**	**III:4**	**III:8**	**III:12**	**IV:3**	**IV:15**
Cataract	Yes	Yes	Yes	Yes	Yes
Morphology	N/A	Mild lamellar + nuclear	N/A	Lamellar + nuclear with prominent sutures and white dots	Mild nuclear with cortical blue dots
Age and Diagnosis	20	22	11	4	9
Age at surgery	41	Not yet operated	19	16	17
Other ocular problems	None	None	Glaucoma	Astigmatism	Myopic astigmatism
Dental features	Edentulous	Pyramidal incisors	Crowding, pyramidal incisors	Crowding, orthodontic work	Crowding, orthodontic work
Other features	None	None	High arched palate	None	High arched palate, narrow face, maxillary hypoplasia, prominent ears

All males show a cataract of similar morphology with surgery in late teens or adulthood. A range of dental and mild morphological features are also observed.

### Genetic analysis

**Figure 3:**
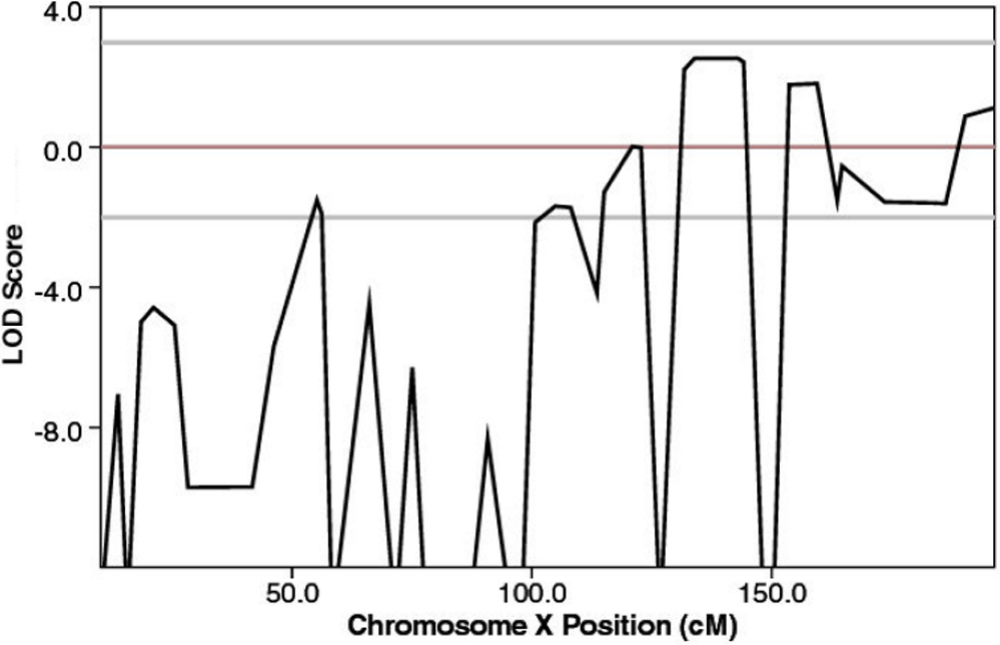
Multipoint linkage analysis conducted in MERLIN. The maximum LOD score obtained was 2.53 for marker DXS1001 and for surrounding markers.

**Figure 4:**
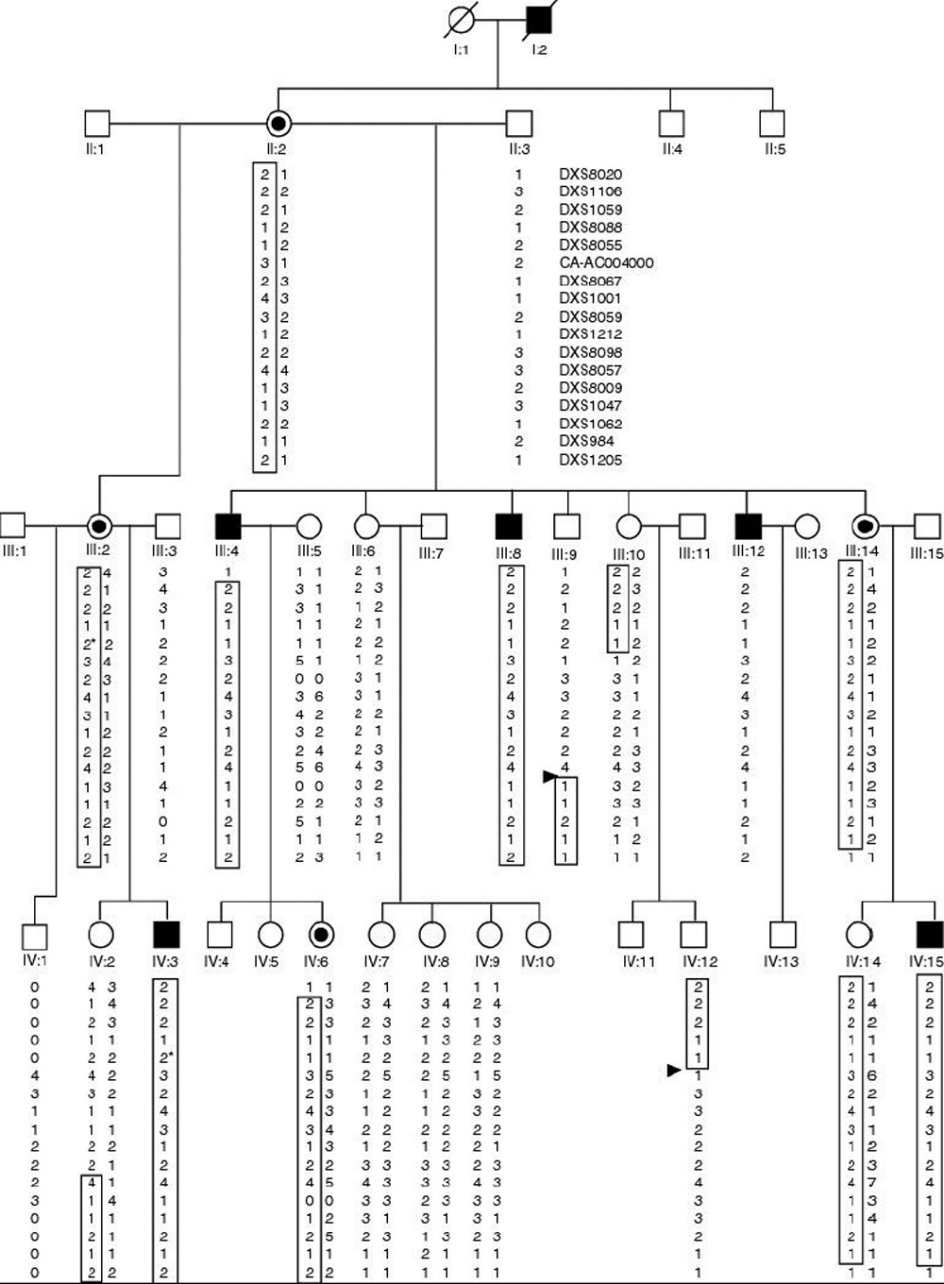
Pedigree of three-generation family. Females are represented by circles and males by squares. Ophthalmologist-confirmed affected individuals are colored black. Obligate carrier females are indicated by a black dot. Haplotypes of all genotyped individuals in the Xq24 region are shown with critical recombination events indicated by an arrowhead. The segregating haplotype is boxed. The alleles marked with an asterisk may represent a mutational event in female III:2 at marker DXS8055.

Significant evidence for linkage at Xq24 was obtained with markers from the initial scan with the maximum two-point and multipoint LOD score of 2.53 for DXS1001 at θ=0 (Table 2 and Figure 3). Equivocal LOD scores were obtained at markers adjacent to this region in both the two-point and multipoint analyses, but inspection of the alleles indicated incomplete segregation at these markers (DXS1059, DXS8088, DXS1062, and DXS984; Figure 4). Additional markers in this region were then genotyped (Table 2), and LOD scores were obtained for informative markers in the region. The peak LOD scores remained at θ=0 for marker DXS1001 with supporting evidence from surrounding markers. No other regions of linkage were detected on the X chromosome (Figure 3). Critical recombination events in individuals III:9 and IV:12 limit the region of interest to 11.5 Mb between markers, DXS8055 and DXS8009 (Figure 4). The recombination event in III:9 indicates that the critical region lies centromeric to marker DXS8055 while the recombination event inherited by IV:12 defines the distal marker as DXS8009. Both critical recombination events occur in unaffected individuals but are observed in males where the phenotype is clear. In addition, a recombination in individual IV:2 suggests that the disease gene may lie centromeric to DXS8057, possibly narrowing the region to 8.9 Mb. However, this individual is female and has no offspring, and thus her carrier status remains to be clarified. Individual III:2 and her affected male offspring do not carry the disease allele at DXS8055 but do at markers on either side, indicating either an unlikely double recombination event or a mutation in this microsatellite.

An attempt was made to further localize the proximal recombination event in III:9 and III:10 between DXS8055 and DXS8067. The marker DXS8064 was included in the original scan but was completely uninformative in this family. A novel dinucleotide repeat between these two markers was genotyped (CA-AC004000) and indicated that the recombination event in both individuals occurred proximally to the novel marker. Therefore, the critical region was not reduced. Similarly, for the recombination in IV:12, four additional markers were typed (DXS8059, DXS1212, DXS8098, and DXS8057), but the recombination occurred telomeric to DXS8057 and the critical region was again not reduced.

**Table 2:** LOD scores at markers on Xq24 at a range of recombination values (θ).

	**Recomination values (θ)**					
**Marker**	**0**	**0.05**	**0.1**	**0.2**	**0.3**	**0.4**
DXS8020	-infin	0.22	0.4	0.43	0.31	0.14
DXS1106	0.43	0.4	0.38	0.31	0.23	0.13
DXS1059	1.33	1.22	1.1	0.83	0.52	0.19
DXS8088	1.33	1.22	1.1	0.83	0.52	0.19
DXS8055	-infin	−0.51	−0.06	0.21	0.21	0.1
DXS8064	0.6	0.56	0.51	0.41	0.29	0.16
CA-AC004000*	2.53	2.31	2.08	1.58	1.04	0.45
DXS8067	2.24	2.02	1.79	1.3	0.78	0.27
DXS1001	2.53	2.31	2.08	1.59	1.04	0.45
DXS8059*	2.53	2.32	2.1	1.62	1.07	0.47
DXS1212*	1.93	1.73	1.52	1.09	0.62	0.19
DXS8098*	0.13	0.12	0.12	0.11	0.08	0.05
DXS8057*	0.73	0.68	0.63	0.52	0.38	0.21
DXS8009	-infin	0.73	0.83	0.69	0.42	0.14
DXS1047	-infin	0.49	0.63	0.61	0.42	0.17
DXS1062	0.12	0.12	0.12	0.11	0.08	0.05
DXS984	0.43	0.4	0.38	0.31	0.23	0.13
DXS1205	-infin	0.19	0.34	0.33	0.2	0.06

Maximum LOD scores of 2.53 were observed at markers CA-AC004000, DXS1001 and DXS8057. The asterisk indicates a marker used for fine mapping the critical recombination events.

## Discussion

We describe a three-generation pedigree with X-linked juvenile cataract displaying recessive inheritance. This pedigree differs from previously reported X-linked pedigrees in that the female carriers have no significant lens opacity. The pedigrees of Fraccaro et al. [1], Krill et al. [2], and Francis et al. [3] all display X-linked codominance with a detectable but milder phenotype in carrier females. In addition, the male phenotype in the reported pedigree is somewhat milder than previously reported X-linked pedigrees with a much later age of diagnosis and cataract extraction not necessary until late teens and beyond. These features suggest a different etiology to the previously described families and indicate the likely presence of a novel locus for X-linked cataract.

The phenotype has been mapped to 11.5 Mb on chromosome X between DXS8055 and DXS8009 by critical recombination events in two unaffected individuals. One must take care when assigning genetic loci based on unaffected status due to the possibility of reduced penetrance. However, the cataract phenotype in males in this pedigree is sufficiently severe that the affection status can be confidently assigned after careful clinical examination. The centromeric recombination event actually occurred in a female. However, this recombined chromosome was passed to her son who is clearly unaffected. Marker DXS8055 is excluded from linkage by the recombination event in III:8. This marker also fails to segregate in female III:2 and her affected son, although markers on either side do segregate. This is more likely to represent a mutational event in the microsatellite rather than a double recombination, which is unlikely over the small genetic distances involved.

In addition to the cataract, affected males also display some mild dysmorphic features and dental anomalies, which may be linked to the cataract phenotype that defines this pedigree. However, these features are quite variable and it is not certain if they are features of a syndrome or coincidental. X-linked cataract-dental syndrome also known as Nance-Horan Syndrome (NHS; OMIM 302350) has been well documented, and the gene on Xp22 has been identified [12]. Similarities between NHS and the phenotype in this family superficially include X-linked cataract and dental anomalies. However, the two phenotypes are quite distinct. Males with NHS display total nuclear cataract in the first few years of life, requiring extraction for normal vision. They also have screwdriver-blade shaped incisors, and many suffer from mental retardation. Carrier females usually display an opacity of the posterior Y-suture, easily detectable on slit lamp biomicroscopy [16]. Both the ocular and dental features in the current family are distinct from this description, and females do not display any detectable phenotype. In addition, no developmental delay has been noted in the affected males. Therefore, this family represents a phenotype distinct from NHS. This conclusion is supported by the linkage analysis excluding linkage to Xp22. Similarly, linkage is excluded from Xp11 where the *MCOPS2* gene has been located. This syndrome also displays cataract and dental anomalies as well as facial and cardiac abnormalities [8].

The gene for the novel phenotype described herein has been localized to an 11.5 Mb region between microsatellite markers, DXS8055 and DXS8009. There are 45 annotated genes in this region, 38 of which are named. Fourteen of these 45 genes have not been detected in the eye-based cDNA libraries reported through the NCBI Unigene database or NEIbank websites and are therefore less attractive as candidates for this phenotype. The remaining 31 genes have been detected in a range of human eye tissues including whole eye, and six of these (*PLS3*, *PGRMC1*, *SEPT6*, *RPL39*, *NDUFA1*, and *LAMP2*) have been reported specifically in lens libraries. The absence of a report in lens does not exclude the gene as a candidate as these collections are not exhaustive. However, the presence of clones of these genes in lens libraries does make them more attractive candidates. One functional candidate, although not previously reported in lens specifically, is the *ZBTB33* gene, which encodes the Kaiso protein. Kaiso interacts with catenin-delta, also known as p120(ctn) [17]. Catenin-delta is a component of cell junctions and has been shown to be present in junction-associated protein complexes of the lens fiber cells [18]. However, direct sequencing of coding regions of the *ZBTB33* gene did not reveal any mutations (data not shown). Regulatory mutations have not been excluded.

Further fine mapping using single nucleotide polymorphisms (SNPs) may be possible if suitably heterozygous markers are identified. However, even in the best case scenario, only eight genes could theoretically be excluded from within the ambiguous areas (DXS8055 to CA-AC004000 and DXS8057 to DXS8099). Of these eight potentially excludable genes, only *PLS3* is known to be expressed in the lens. With the ever reducing cost of sequencing in today’s genomics environment, a candidate gene approach is now being pursued rather than additional fine mapping.

The late onset of clinically significant cataract indicates that the gene is not a major player in fetal development of the lens but is present in the adult lens. These clues will help narrow the candidate gene list as more information on the temporal and physical expression pattern of genes in the critical region is gathered. The eventual identification of the causative gene may help elucidate the process of adult cataractogenesis.

## Acknowledgments

This research was supported by project grant number 275566 from the National Health and Medical Research Council of Australia (NHMRC). J.E.C. is supported by a practioner fellowship from the NHMRC and KPB is and NHMRC Peter Doherty Fellow.
